# Prognostic impact and timing considerations for allogeneic hematopoietic stem cell transplantation in chronic myelomonocytic leukemia

**DOI:** 10.1038/s41408-020-00387-y

**Published:** 2020-11-20

**Authors:** Prateek Pophali, Aasiya Matin, Abhishek A. Mangaonkar, Ryan Carr, Moritz Binder, Aref Al-Kali, Kebede H. Begna, Kaaren K. Reichard, Hassan Alkhateeb, Mithun V. Shah, Ayalew Tefferi, William J. Hogan, Mark R. Litzow, Mrinal M. Patnaik

**Affiliations:** 1 Division of Hematology, Department of Medicine, Mayo Clinic, Rochester, Minnesota USA; 2 Division of Hematopathology, Department of Pathology, Mayo Clinic, Rochester, Minnesota USA

**Keywords:** Myelodysplastic syndrome, Chronic myeloid leukaemia

**Dear Editor,**

Chronic myelomonocytic leukemia (CMML) is a clonal disorder of aging hematopoietic stem cells characterized by overlapping features of myeloproliferation and myelodysplasia^1^, with a median overall survival (OS) of ≤36 months^1,2^. Hypomethylating agents (HMAs) have an overall response rate of about 30–40%; however, these agents are ineffective in altering the natural disease biology due to inability to prevent acquisition of molecular abnormalities and transformation to acute myeloid leukemia (AML)^3^. Allogeneic hematopoietic stem cell transplantation (alloHCT) is a potentially curative option, with 20–50% patients achieving long-term remissions. However, as the median age of presentation is 73 years, only a fraction (<20%) of CMML patients are eligible for alloHCT^4^. Prognostic models such as the Mayo Prognostic Model (MPM), Mayo Molecular Model (MMM), Groupe Francais des Myelodysplasies, and the CMML-specific prognostic scoring system (CPSS) are important tools to identify patients at high risk for disease progression and death^5–9^. Several retrospective analyses of outcomes in alloHCT patients have identified adverse cytogenetics, blast percentage, HCT-comorbidity Index, time to alloHCT, disease control at the time of alloHCT and acute and chronic graft vs. host disease (GVHD) as factors influencing OS and AML-free survival (LFS)^10–12^^,14,15^.

In the absence of randomized controlled trials, the questions of optimal timing of alloHCT in CMML, pre-alloHCT use of HMA vs. cytotoxic chemotherapy, and the selection of patients who should be treated upfront with alloHCT remain unanswered. We performed this study to assess the outcomes and therapeutic impact of alloHCT in patients with CMML.

## Study population

After Mayo Clinic Institutional Review Board approval, medical records of 406 consecutive CMML patients (age ≤ 75 years at diagnosis) from January 1990 to December 2018 were reviewed (75 years being the upper age limit for alloHCT in our institution). Disease and alloHCT-related data were retrospectively collected. Next-generation sequencing (Supplementary Table 1S) for myeloid-relevant mutations was performed on bone marrow mononuclear cells at CMML diagnosis, or at first referral (within 6 months of diagnosis). Response assessment was documented as per the 2015 International Working Group (IWG) myelodysplastic/myeloproliferative neoplasm overlap neoplasm criteria^13^. The CPSS, MPM, MMM, and the Mayo-French Models (MFMs) were employed for risk stratification. A 1:1 propensity score matching (PSM) analysis for age and MPM risk stratification (low, intermediate, and high risk) was used to determine the impact of alloHCT in patients who did and did not undergo alloHCT. High-risk cytogenetics included complex and monosomal karyotypes, and low risk included normal, sole -Y, and sole der (3q), with the rest being included under the intermediate category per the MFM model^9^. Kaplan–Meier estimate of OS was computed from date of diagnosis to date of death or censored at last follow-up. In LFS calculation, AML replaced death as the uncensored event (details in Supplementary File under section “Supplementary Methods”).

## Clinico-pathologic characteristics

Seventy (17%) CMML patients with a median age at diagnosis of 58 (range: 18–73) years underwent alloHCT at our institution; 45 (64%) males, 46 (66%) in chronic phase (CP), and 24 (34%) after AML/blast transformation (BT) (Table 1). In the non-alloHCT (control) group, we identified 336 consecutive CMML patients with age at diagnosis ≤ 75 [median 67 (range: 20–75)] years, 267 (79%) patients in CP, and 64 (19%) with CMML that eventually had BT. The two groups were evenly matched for molecular abnormalities, except for a lower frequency of *TET2* mutations in the alloHCT group (30% vs. 56%, *p* = 0.02, Supplementary Table 2S).

**Table 1:** Table describing clinico-pathologic and genetic characteristics of chronic myelomonocytic leukemia treated with allogeneic hematopoietic stem cell transplant.

Variables	All CMML patients who underwent HCT (*n* = 70)	CMML patients who underwent HCT in chronic phase (*n* = 46)	CMML patients who underwent HCT after blast transformation (*n* = 24)	*P*-value
Age in years; median (range)	58 (18–73)	58 (26–72)	57 (18–73)	0.24
Sex (Male); *n* (%)	45 (64)	31 (67)	14 (58)	0.45
Hemoglobin g/dL; median (range)	9.5 (6.4–12.5)	9.1 (6.4–12.5)	10 (6.7–12.2)	**0.02**
WBC × 10^9^/L; median (range)	1.6 (0.1–52.1)	1.8 (0.1–52.1)	1.1 (0.1–6)	0.10
ANC × 10^9^/L; median (range)	1.5 (0–43.2)	1.4 (0–43.2)	1.7 (0–5.6)	0.83
Platelets × 10^9^/L; median (range)	56 (7–277)	47 (7–194)	65 (18–277)	0.07
Palpable splenomegaly at transplant; *n* (%)	9 (13)	5 (11)	4 (18)	0.43
Prior therapies; *n* (%)	*N* = 68	*N* = 44	*N* = 24	<**0.0001**
Observation/supportive care	10 (15)	10 (23)	0 (0)	
HMA	22 (32)	21 (48)	1 (4)	
AML-like induction chemotherapy	18 (27)	5 (11)	13 (54)	
HMA followed by induction chemotherapy	12 (18)	2 (4)	10 (42)	
Clinical trial	3 (4)	3 (7)	0 (0)	
Other	3 (4)	3 (7)	0 (0)	
Disease status at transplant; *n* (%)	*N* = 67	*N* = 44	*N* = 23	**0.0004**
Complete remission	23 (34)	8 (18)	15 (65)	
Marrow response	22 (33)	17 (39)	5 (22)	
Stable disease	8 (12)	8 (18)	0 (0)	
Disease progression	14 (21)	11 (25)	3 (13)	
Time to transplant from diagnosis in months; median (range)	5 (0–44)	7 (0–22)	7 (2–44)	0.33
HCT-CI; *n* (%)	*N* = 68	*N* = 45	*N* = 23	0.30
Group 1 (0)	16 (24)	8 (18)	8 (35)	
Group 2 (1–2)	18 (26)	13 (29)	5 (22)	
Group 3 (≥3)	34 (50)	24 (53)	10 (43)	
Type of transplant conditioning; *n* (%)	*N* = 68	*N* = 46	*N* = 22	0.61
Myeloablative	31 (46)	20 (43)	11 (50)	
Reduced intensity	37 (54)	26 (57)	11 (50)	
Donor source; *n* (%)	*N* = 67	*N* = 45	*N* = 22	0.83
Matched related donor	28 (42)	17 (38)	11 (50)	
Mismatched related donor	1 (2)	1 (2)	0 (0)	
Matched unrelated donor	30 (45)	22 (49)	8 (36)	
Mismatched unrelated donor	3 (4)	2 (4)	1 (5)	
Umbilical cord blood	2 (3)	1 (2)	1 (5)	
Haploidentical donor	3 (4)	2 (4)	1 (5)	
Graft source; *n* (%)	*N* = 68	*N* = 45	*N* = 23	0.76
Peripheral blood	58 (85)	38 (85)	20 (87)	
Bone marrow	8 (12)	6 (13)	2 (9)	
Umbilical cord	2 (3)	1 (2)	1 (4)	
Acute GVHD grade 2–4; *n* (%)	29/63 (46)	17/43 (40)	12/20 (60)	0.12
Chronic GVHD; *n* (%)	*N* = 41	*N* = 30	*N* = 11	0.24
Mild	15 (37)	13 (43)	2 (18)	
Moderate	14 (34)	10 (33)	4 (36)	
Severe	12 (29)	7 (23)	5 (45)	
GVHD-free, relapse-free survival in months; median (95% CI)	6 (5–8)	7 (5–21)	3.5 (2–7)	**0.02**
GRFS at 100 days post HCT in percentage	70	76	54	
Posttransplant disease relapse; *n* (%)	18 (27)	11 (24)	7 (33)	0.45
Overall survival post transplant; median (95% CI)	25 (18–189)	67 (20–189)	16 (7–39)	0.06
Deaths	39 (56)	22 (48)	17 (71)	0.06

Of the 46 patients transplanted in CP, 31 (67%) patients received prior therapies such as HMA (48%), AML-like induction chemotherapy (11%), or investigational agents (7%). Of the 24 patients transplanted in BT, 13 (54%) patients received prior AML-like induction and 10 (42%) received HMA followed by induction chemotherapy prior to alloHCT. There was no statistically significant difference in day 100 mortality in patients who received pre-transplant HMA vs. induction therapy in both CP (*p* = 0.5) and BP (*p* = 0.1) CMML patients. Of the 70 CMML patients who received alloHCT, only 7 were untreated prior to receiving conditioning therapies. There was no difference in Kaplan–Meier estimate of median OS in untreated patients vs. those who received pre-transplant cytoreduction (HMA, AML-like induction therapy, and investigational agents; log-rank test, *p* = 0.3). Twenty five (57%) and 20 (87%) patients transplanted in CP and BT, respectively, met criteria for complete response (CR) or optimal marrow response at the time of alloHCT. The conditioning regimens (myeloablative vs. reduced intensity) and donor sources (matched vs. mismatched, related vs. unrelated) were evenly matched in both the CP- and BT-transplanted CMML patients (Table 1). Peripheral blood stem cells were the favored donor source in both groups (85% in CP vs. 87% in BT, *p* = 0.76). Sixty six (94%) patients had sustained donor engraftment. Similarly, rates of acute (40% vs. 60%, *p* = 0.12) and chronic (65% vs. 46%, *p* = 0.24) GVHD were not significantly different between CMML patients transplanted in CP and BT (Table 1). None of the transplanted patients received posttransplant HMA therapy.

## Survival outcomes

At a median follow-up of 70 (95% confidence interval (95% CI) 27–189) months, there were 22 (31%) deaths in the CP alloHCT group; 11 (24%) from disease relapse, and 9 (20%) from non-relapse mortality. Four (9%) died from infections, 2 (4%) from acute GVHD, and 2 (4%) from multi-organ failure (1-unknown). Median OS was the higher in the CP vs. BT alloHCT group [70 (95% CI 27–189) vs. 32 (95% CI 15–59) months, *p* = 0.001, Supplementary Fig. 1S] and lower in non-alloHCT group [29 (95% CI 23–35) months in CP and 22 (95% CI 14–31) months in BT-CMML]. Post-alloHCT median OS was 67 (95% CI 20–189) months in CP and 16 (95% CI 7–39) months in BT-CMML (*p* = 0.06; Supplementary Fig. 2S). Median OS in transplant-eligible CMML BT patients (≤75 years old) who received alloHCT after BT was 22 months vs. 3 months in the non-alloHCT group (Supplementary Fig. 3S). Five-year OS in the post-alloHCT group was 51% in the CP and 19% in the BT (Supplementary Table 3S). Similar to median OS, median LFS in the non-alloHCT group was lower compared to alloHCT group [24 (95% CI 20–28) months vs. 59 (95% CI 27–189) months, *p* = 0.0001; Supplementary Fig. 4S]. BT patients continued to fare poorly in the alloHCT group with a median LFS of 7.5 months vs. 56 months in the CP alloHCT group (*p* = 0.01, Supplementary Fig. 5S). At 5 years, the LFS was 47% in the CP patients who underwent alloHCT vs. only 12% who underwent alloHCT in BT (Supplementary Table 4S). At a median follow-up of 32 (95% CI 15–59) months in the BT alloHCT group, there were 17 (22%) deaths: 6 (25%) from disease relapse, 4 (13%) due to GVHD (2 each from acute and chronic GVHD), 5 (21%) due to infection, and 2 (8%) from multi-organ failure (Supplementary Tables 5S and 6S).

Among the CP alloHCT recipients, median OS was not reached in the CPSS intermediate 1 (95% CI 3-NR) and 2 (95% CI 21-NR) risk groups, and was 12 (95% CI 2–67, *p* = 0.02) months in the high-risk group (Supplementary Fig. 6S). Likewise, post-alloHCT median OS was not reached in the intermediate- and low-risk MPM categories in CP patients, vs. a post-alloHCT median OS of 36 (95% CI 8–189) months in high-risk patients (Supplementary Fig. 7S). These posttransplant OS trends were mirrored when CP patients were risk stratified as per the MFM (Supplementary Fig. 8S).

The composite end point of median GVHD-free/relapse-free survival was 3.5 (95% CI 2–7) and 7 (95% CI 5–21) months in the BT and CP, respectively [*p* = 0.02, Supplementary Table 7S]. Further, LFS and OS in the alloHCT group did not differ in CMML alloHCT patients with vs. without chronic GVHD (Supplementary Table 8S). In the transplant eligible (age ≤ 75 years) group (*n* = 406), 200 (49%) patients were classified as proliferative CMML, whereas 203 (50%) were classified as dysplastic CMML (information not available for 3 patients). The Kaplan–Meier estimate median OS in the proliferative subtype was significantly lower when compared to the dysplastic subtype (20 vs. 32 months, log-rank *p* < 0.001). In the proliferative subtype, the median OS for transplant group was higher than the non-transplant group (50 vs. 19 months, log-rank *p* < 0.0001). In dysplastic subtype, the median OS for transplant and non-transplant group was not significantly different (41 vs. 37 months, log-rank *p* = 0.5). The Kaplan–Meier estimate of median OS in patients with CR or optimal pre-transplant blast % (defined as BM blast% < 5) was higher than those without CR or optimal blast% (50 vs. 27 months); however, this difference was not statistically significant (log-rank test, *p* = 0.2).

## Survival analysis

In a univariate survival analysis that included age, sex, CMML prognostic models, cytogenetic abnormalities, gene mutations, pre-alloHCT therapy, remission status at alloHCT, HCT-CI, time to alloHCT, donor types, stem cell source, human leukocute antigen matching, conditioning regimen, cytomegalovirus (CMV) status, blood group incompatibility, pre-alloHCT complete blood count (CBC), acute and chronic GVHD, only abnormal karyotype (MFM intermediate and high risk; hazard ratio (HR) 2.63, 95% CI 1.11–6.23, *p* = 0.03) adversely impacted outcomes in CP-CMML patients that underwent alloHCT. In univariate analysis for post-alloHCT CP-CMML LFS, abnormal karyotype (MFM intermediate and high risk; 2.78, 95% CI 1.18–6.58, *p* = 0.02), WBC < 2 × 10^9^/L at time of alloHCT (1.06, 95% CI 1.01–1.11, *p* = 0.01), and absolute neutrophil count (ANC) < 1.5 × 10^9^/L at the time of alloHCT (1.08, 95% CI 1.02–1.13, *p* = 0.01) were adverse predictors. In the entire cohort of transplant-eligible CMML patients (including CP-CMML and BT-CMML patients), the three most common mutations were *ASXL1* (55%), *TET2* (52%), and *SRSF2* (46%). In CP-CMML patients, the frequency of these mutations were *ASXL1* 55%, *SRSF2* 47%, and *TET2* 35%, whereas in BT-CMML patients, the frequencies were *ASXL1* 44%, *SRSF2* 43%, and *TET2* 30%. In a univariate survival analysis, none of the mutations predicted post-HCT outcomes (Supplementary Tables 9S and 10S).

On multivariate analysis, only abnormal karyotype (MFM intermediate and high risk; HR 2.73, 95% CI 1.04–7.17, *p* = 0.02) retained its negative prognostic impact (Supplementary Tables 9S and 10S).

## Propensity score matched analysis

**Fig. 1:**
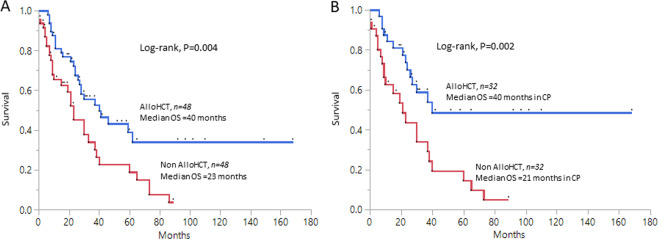
Figure showing Kaplan–Meier derived overall survival curves among propensity score matched chronic myelomonocytic leukemia (CMML) cohorts. **a** Overall survival in a 1:1 propensity score matched cohort for age and Mayo Prognostic Model in allogeneic hematopoietic stem cell transplant (alloHCT) and non-alloHCT groups [inclusive of patients in chronic phase (CP) and blast transformation (BT)]. **b** Overall survival in a 1:1 propensity score matched cohort for age and Mayo Prognostic Model in alloHCT and non-alloHCT groups in CP only.

Forty-eight patients in alloHCT and non-alloHCT groups were matched for age and MPM using 1:1 PSM analysis. Median OS in the PSM-matched alloHCT group was higher compared to non-alloHCT group [40 months, (95% CI 26–NR) vs. 23 months, (95% CI 10–37), *p* = 0.004, Fig. 1a]. When only CP-CMML alloHCT and non-alloHCT (*n* = 32) 1:1 matched patients were considered, the survival advantage remained significant in the alloHCT group [40 months, (95% CI 26–NR) vs. 21 months (95% CI 9–40), *p* = 0.002, Fig. 1b]. Similarly, CP alloHCT group had a higher LFS vs. CP non-alloHCT PSM-matched group [40 (95% CI 26–NR) vs. 20 (95% 9–40) months, *p* = 0.002].

In summary, within limitations of a retrospective analysis, our study confirms the survival benefit conferred by alloHCT in CMML, especially in CP disease. AlloHCT was able to achieve a 5-year OS of 51% in CP-CMML vs. 19% in BT-CMML, underscoring the importance of early alloHCT, especially in higher risk patients. This observation was also validated with the help of a propensity score-based comparison (Fig. 1b). The survival advantage of alloHCT was somewhat offset by a GFRFS of only 7 months, indicating that in CMML, alloHCT can be associated with significant morbidity. We also show that intermediate to high-risk cytogenetic abnormalities by MFM are predictive of post-alloHCT relapse and inferior OS, highlighting the need for better pre-alloHCT therapies.

## Supplementary information

Supplement material
