# Supplementary material for: Prognostic impact and timing considerations for allogeneic hematopoietic stem cell transplantation in chronic myelomonocytic leukemia
Source: Blood Cancer J. 2020 Nov 20;10(11):121. doi: 10.1038/s41408-020-00387-y (PMC7679455; doi:10.1038/s41408-020-00387-y)
Supplement: Supplementary file 1 — Supplement material [file 41408_2020_387_MOESM1_ESM.docx]

**Supplementary Material**

**Supplementary table legends:**

Table 1S: Next Generation Sequencing panel details to detect myeloid-relevant mutations.

Table 2S: Patient related characteristics of all CMML patients stratified by those who were treated versus untreated with alloHCT.

Table 3S: Table showing post alloHCT OS in CMML patients transplanted at CP and BT.

Table 4S: AML-free survival in alloHCT patients in CP and BT phase.

Table 5S: Incidences of disease relapse after alloHCT in CMML patients transplanted in CP and BT phase.

Table 6S: Treatment and non-relapse mortality in CMML CT and BT groups post alloHCT.

Table 7S: GVHD-free/relapse-free (GRFS) survival in CMML patients who underwent alloHCT in both CP and BT phase.

Table 8S: Post alloHCT OS and LFS in CMML alloHCT patients with versus without chronic graft versus host disease (cGVHD).

Table 9S: Univariate and multivariate post-transplant OS analysis of 46 patients who underwent allogeneic hematopoietic cell transplantation in chronic phase CMML.

Table 10S: Univariate and Multivariate post-transplant LFS analysis of 46 patients who underwent allogeneic hematopoietic cell transplantation in chronic phase CMML.

**Supplementary figure legends:**

Figure 1S: Overall Survival in CMML patients in CP, BT alloHCT and who did not undergo alloHCT.

Figure 2S: Post alloHCT median OS in CMML patients who underwent alloHCT in CP versus BT.

Figure 3S: Median OS in patients (age < 75 years) with BT who underwent alloHCT vs non-alloHCT.

Figure 4S: AML-free survival in CMML CP alloHCT versus non alloHCT cohort.

Figure 5S: AML-free survival in alloHCT patients in CP and BT.

Figure 6S: Post alloHCT OS by CMML specific prognostic scoring system (CPSS) categories in CMML patients in chronic phase.

Figure 7S: Post alloHCT OS by Mayo Prognostic Model category in CMML chronic phase patients.

Figure 8S: Post alloHCT OS by Mayo French Model category in CMML chronic phase patients.

| **Table 1S: Next Generation Sequencing panel details to detect myeloid-relevant mutations** | | | | |
| --- | --- | --- | --- | --- |
| Gene panel | Institution | List of genes | Coding region coverage | Read depth |
| OncoHeme NGS for Hematologic Cancers | Mayo Clinic | *ASXL1; BCOR; BRAF; CALR; CBL; CEBPA; CSF3R; DNMT3A; ETV6; EZH2; FLT3; GATA1; GATA2; IDH1; IDH2; JAK2; KIT; KRAS; MPL; MYD88; NOTCH1; NPM1; NRAS; PHF6; PTPN11; RUNX1; SETBP1; SF3B1; SRSF2; TERT; TET2; TP53; U2AF1; WT1; ZRSR2* | Variable per gene: see details at <https://www.mayomedicallaboratories.com/test-catalog/Overview/63367> | >250X |
| Research NGS | Mayo Clinic | *ASXL1; ASXL2; ATM; BCOR; BCORL1; CALR; CBL; CEBPA; CSF3R; DNMT3A; EED; ETNK1; EZH2; FLT3; GATA2; IDH1; IDH2; JAK2; JARID2; KIT; KRAS; MPL; NRAS; PHF6; PTPN11; RPS6KA2; RUNX1; SETBP1; SF3B1; SH2B3; SRSF2; STAG2; STK11; SUZ12; TERC; TERT; TET1; TET2; TET3; TP53; U2AF1; ZRSR2* | Full exon region coverage for each gene. Overall *TET2* coverage was low in some cases (<12X) and therefore unable to interpret. | >500X |

| **Table 2S: Patient related characteristics of all CMML patients stratified by those who were treated versus untreated with alloHCT.** | | | | |
| --- | --- | --- | --- | --- |
| Variables | **All CMML patients aged**  **< 75**  **(n=406)** | **CMML patients who underwent HCT**  **(n=70)** | **CMML patients who did not undergo HCT**  **(n=336)** | **P value** |
| Age in years; median (range) | 66 (18-75) | 57 (18-73) | 67 (20-75) | **<0.0001** |
| Males; *n* (%) | 275 (67.7) | 45 (64) | 230 (68) | 0.50 |
| Hemoglobin, g/dL; median (range) | 11 (4.3-17) | 10.6 (4.3-17) | 11 (5.2-16.8) | **0.03** |
| WBC x10^9^/L; median (range) | 13.3 (1.1-180) | 14.3 (1.1-102) | 13.1 (1.3-180) | 0.31 |
| ANC x 10^9^/L; median (range) | 6.7 (0-151) | 6.8 (0.3-81.6) | 6.4 (0-151) | 0.35 |
| AMC x 10^9^/L; median (range) | 2.7 (0-84) | 2.8 (0-43.1) | 2.6 (0.1-84) | 0.65 |
| ALC x 10^9^/L; median (range) | 2 (0-14.5) | 2.5 (0.22-12) | 1.9 (0-14.5) | **0.02** |
| Platelets x 10^9^/L; median (range) | 101 (7-1277) | 105 (16-587) | 100.5 (7-1277) | 0.63 |
| Presence of circulating immature myeloid cells; *n* (%) | 252 (63) | 52 (74) | 200 (61) | **0.03** |
| PB blast %; median (range) | 0 (0-18) | 1 (0-15) | 0 (0-18) | **<0.0001** |
| BM blast %; median (range) | 3 (0-19) | 5 (0-18) | 3 (0-19) | **<0.0001** |
| 2016 WHO morphologic subtypes; *n* (%)  CMML-0  CMML-1  CMML-2 | 218 (54)  105 (26)  80 (20) | 21 (30)  24 (34)  25 (35) | 197 (59)  81 (24)  55 (17) | **<0.0001** |
| Abnormal cytogenetics; *n* (%) | 116/385 (30) | 24/70 (34) | 92/315 (29) | 0.40 |
| Mayo-French cytogenetic risk stratification; *n* (%)  Low  Intermediate  High | 279 (73)  74 (19)  31 (8) | 46 (67)  13 (19)  10 (14) | 233 (74)  61 (19)  21 (7) | 0.13 |
| Mayo prognostic model; *n* (%)  Low  Intermediate  High | 53 (13)  145 (37)  197 (50) | 5 (7)  24 (35)  40 (58) | 48 (15)  121 (37)  157 (48) | 0.14 |
| Next generation sequencing analysis; *n* (%)   1. Epigenetic regulators   *TET2*  *DNMT3A*  *IDH1*  *IDH2*   1. Chromatin regulators   *ASXL1*  *EZH2*   1. Transcription factors   *RUNX1*  *BCOR*   1. Spliceosome components   *SF3B1*  *SRSF2*  *U2AF1*  *ZRSR2*   1. Cell signaling regulators   *JAK2 V617F*  *MPL*  *CALR*  *CBL*  *KRAS*  *NRAS*  *PTPN11*  *CSF3R*  *C-KIT*  *FLT3TKD*   1. Tumor suppressor genes   *Tp53*   1. Others   *SETBP1* | N=229  119 (52)  13 (6)  2 (1)  13 (6)  133 (55)  11 (5)  28 (12)  2 (1)  8 (3)  105 (46)  19 (8)  7 (3)  22 (10)  1 (0.5)  0 (0)  35 (15)  14 (6)  36 (16)  8 (3)  3 (1)  11 (5)  7 (3)  9 (4)  31 (13) | N=33  10 (30)  1 (3)  0 (0)  2 (6)  19 (58)  2 (6)  3 (9)  0 (0)  0 (0)  12 (36)  5 (15)  1 (3)  2 (6)  0 (0)  0 (0)  4 (12)  2 (6)  6 (18)  1 (3)  0 (0)  1 (3)  1 (3)  2 (6)  3 (9) | N=196  109 (56)  12 (6)  2 (1)  11 (6)  114 (54)  9 (5)  25 (13)  2 (1)  8 (4)  93 (47)  14 (7)  6 (3)  20 (10)  1 (0.5)  0 (0)  31 (16)  12 (6)  30 (15)  7 (4)  3 (2)  10 (5)  6 (3)  7 (4)  28 (14) | **0.02**  0.94  0.42  0.97  0.75  0.35  0.84  0.43  0.10  0.15  0.07  0.96  0.39  0.57  .  0.51  0.59  0.89  0.84  0.32  0.55  0.96  0.56  0.35 |
| Molecular Mayo model; *n* (%)  Low  Intermediate-1  Intermediate-2  High | 19 (8)  56 (24)  78 (33)  85 (36) | 0 (0)  5 (15)  11 (33)  17 (52) | 19 (9)  51 (25)  67 (33)  68 (33) | **0.02** |
| Blast transformation; *n* (%) | 93 (23) | 29 (41) | 64 (19) | **0.0002** |
| Deaths; *n* (%) | 260 (64) | 221 (66) | 39 (56) | 0.11 |
| Follow-up, in months; median (range) | 65 (1-233) | 60 (6-189) | 80 (1-233) | 0.31 |

The bold values represent p values that are statistically significant; *p* < 0.05.

**Key:** ALC: absolute lymphocyte count; AMC: absolute monocyte count; ANC: absolute neutrophil count; WBC: white blood cell count; PB: peripheral blood; BM: bone marrow; WHO: World Health Organization.

No HCT ≤75 yrs

HCT in CP

HCT after BT

*n=46*

Median OS=70 months

*n=336*

Median OS=27 mo

*n=24,* Median OS=32mo

Log-rank P=0.0014

**Figure 1S: Overall Survival in CMML patients in CP, BT alloHCT and who did not undergo alloHCT**

Log-rank P=0.06

P=0.002

CP alloHCT, *n=46*

Post alloHCT Median OS= 67 mo

BT alloHCT, *n=24*

Post alloHCT Median OS= 16 mo

**Figure 2S: Post alloHCT median OS in patients who underwent alloHCT in CP versus BT**

Log-rank P=0.0001

AlloHCT, *n= 24*

Median OS = 22 months

Non-AlloHCT, *n= 63,* Median OS = 3 months

**Figure 3S: Median OS in patients (age < 75 years) with BT who underwent alloHCT vs non-alloHCT**

**Table 3S: Table showing post alloHCT OS in CMML patients transplanted at CP and BT**

|  | Post-HCT in Overall Survival | Post-HCT OS in CP | Post-HCT OS in BT |
| --- | --- | --- | --- |
| 1 year OS | 65% | 71% | 54% |
| 3 year OS | 45% | 51% | 35% |
| 5 year OS | 40% | 51% | 19% |

Log- rank P=0.0001

*n=46*, LFS= 59 months

*n=329*, LFS= 24 months

**Figure 4S: AML-free survival (LFS) in CMML CP alloHCT versus non alloHCT cohort.**

Log-rank P=0.01

AlloHCT in CP, *n=46*

Median LFS = 56 months

AlloHCT in BT, *n=24*

Median LFS = 7.5 months

**Figure 5S: AML-free survival in alloHCT patients in CP and BT.**

**Table 4S: AML-free survival in alloHCT patients in CP and BT phase.**

|  | **Overall LFS** | **LFS HCT in CP** | **LFS HCT post BT** |
| --- | --- | --- | --- |
| 1 year LFS | 58% | 67% | 42% |
| 3 year LFS | 45% | 52% | 24% |
| 5 year LFS | 36% | 47% | 12% |

**Table 5S: Incidences of disease relapse after alloHCT in CMML patients transplanted in CP and BT phase.**

|  | **Overall** | **HCT in CP** | **HCT post LT** | **P value** |  | **RIC** | **MAC** | **P value** |
| --- | --- | --- | --- | --- | --- | --- | --- | --- |
| Relapse rate; n (%) | 18 (26) | 11 (24) | 7 (29) | 0.63 |  | 11 (30) | 7 (23) | 0.50 |

**Table 6S: Treatment and non-relapse mortality in CMML CT and BT groups post alloHCT.**

|  | **Overall** | **HCT in CP** | **HCT post LT** |  | **RIC regimen** | **MA regimen** |
| --- | --- | --- | --- | --- | --- | --- |
| **Treatment related mortality** | | | | | | |
| **3 months** | 6% | 4% | 8% |  | 5% | 7% |
| **1 year** | 15% | 9% | 25% |  | 5% | 24% |
| **5 years** | 20% | 12% | 32% |  | 5% | 32% |
| **Non relapse mortality** | | | | | | |
| **3 months** | 0% | 0% | 0% |  | 0% | 0% |
| **1 year** | 6% | 3% | 12% |  | 4% | 9% |
| **5 years** | 18% | 17% | 19% |  | 20% | 17% |

Log-rank P=0.02

Intermediate 2, *n=28,* Post alloHCT OS =NR

High Risk, *n=7,*

Post alloHCT OS =12 months

Intermediate 1 Risk, *n=7,*

Post alloHCT OS =NR

**Figure 6S: Post alloHCT OS by CMML specific prognostic scoring system (CPSS) categories in CMML patients in chronic phase.**

Low Risk, *n=2*

Post AlloHCT OS=NR

Log-rank P=0.69

High Risk, *n=27*

Post AlloHCT OS =36 months

Intermediate Risk, *n=15*

Post AlloHCT OS=NR

**Figure 7S: Post alloHCT OS by Mayo Prognostic Model category in CMML chronic phase patients.**

(In months)

High Risk, *n=5*

Post AlloHCT median OS= 21(2-189) months

Intermediate Risk, *n*= 8

Post AlloHCT median OS= 16(3-NR) months

Low Risk, *n=32*

Post AlloHCT median OS=NR (21-NR)

Log-rank P=0.09

**Figure 8S: Post alloHCT OS by Mayo French Model (MFM) category in CMML chronic phase patients.**

**Table 7S: GVHD-free/relapse-free (GRFS) survival in CMML patients who underwent alloHCT in both CP and BT phase.**

|  | **Overall** | **HCT in CP**  **(n=45)** | **HCT post BT**  **(n=24)** | **P value** | **RIC(n=37)** | **MAC (n=31)** | **P value** |
| --- | --- | --- | --- | --- | --- | --- | --- |
| GVHD free relapse free survival in months (95% CI) | 6 (5-8) | 7 (5-21) | 3.5 (2-7) | **0.02** | 6 (3-8) | 7 (4-36) | 0.07 |
| GRFS at 100 day | 70% | 78% | 54% |  | 65% | 74% |  |

**Table 8S: Post alloHCT OS and LFS in CMML alloHCT patients with versus without chronic graft versus host disease (cGVHD).**

| **Minimum survival 100 days** | **Patients with cGVHD** | **Patients without cGVHD** | **P value** |
| --- | --- | --- | --- |
| **Leukemia free survival** | 42 (21-NR) | 11 (5-189) | 0.09 |
| **Overall survival** | 67 (21- NR) | 16 (8-189) | 0.055 |

| **Table 9S: Univariate and multivariate post-transplant OS analysis of 46 patients who underwent allogeneic hematopoietic cell transplantation in chronic phase CMML** | | | | |
| --- | --- | --- | --- | --- |
|  | **Univariate analysis for post-transplant OS** | | **Multivariate analysis for post-transplant OS** | |
| **Variables** | **HR for OS (95% CI)** | **P value** | **HR for OS (95% CI)** | **P value** |
| Age at Transplant | 1.00(0.96-1.045) | 0.96 | - |  |
| Gender (M/F) | 1.16(0.47-2.87) | 0.75 | - |  |
| PB blast %; median (range) | 1.04(0.88 – 1.15) | 0.60 | **-** |  |
| BM blast %; median (range) | 1.05(0.95 – 1.14) | 0.32 | **-** |  |
| WHO FAB subtype  (Dysplastic/proliferative) | 1.15(0.49-2.71) | 0.75 | - |  |
| Mayo prognostic model  (0/1)  (1/2)  (0/2) | 5.20(0-0)  0.99(0.40-2.46)  5.14(0-0) | 0.51 | - |  |
| Mayo molecular model  (1/2)  (2/3)  (1/3) | 6.41(0)  0.56(0.11-2.8)  3.57(0) | 0.25 | - |  |
| Abnormal cytogenetics  (Abnormal/Normal) | **2.63(1.11-6.23)** | **0.03** | - |  |
| Mayo French cytogenetic risk group  (Low/Intermediate)  (Intermediate/High) | 0.39 (0.13-1.24)  1.02 (0.27-3.85) | 0.13 | - |  |
| Mutations:   1. *TET2* 2. *DNMT3A* 3. *IDH1* 4. *IDH2* 5. *ASXL1* 6. *EZH2* 7. *RUNX1* 8. *BCOR* 9. *SF3B1* 10. *SRSF2* 11. *U2AF1* 12. *ZRSR2* 13. *JAK2* 14. *CBL* 15. *KRAS* 16. *NRAS* 17. *CSF3R* 18. *C-KIT* 19. *FLT3* 20. *TP53* 21. *SETBP1* | 0.21 (0.03-1.69)  7.49 (0.77-72.05)  --  --  0.86 (0.20-3.59)  --  --  --  --  0.31 (0.04-2.50)  0.64 (0.08 -5.34)  --  --  --  --  3.75 (0.87-16.09)  --  --  --  4.0 (0.46-34.40)  -- | 0.08  0.15  --  0.50  0.83  0.12  0.15  --  --  0.20  0.66  0.50  0.77  0.85  0.39  0.10  --  0.39  0.39  0.28  0.78 | - |  |
| Therapy related | 0.64 (0.09-4.87) | 0.64 | - |  |
| HMA therapy before transplant | 0.85 (0.34-2.14) | 0.54 | - |  |
| AML induction like chemotherapy before transplant | 1.75 (0.58-5.21) | 0.34 | - |  |
| Disease status at transplant  PD/OMR  OMR/CR | 2.33 (0.73-7.41)  1.31. (0.25-6.79) | 0.36 | - |  |
| HCT CI groups*  3/1  3/2  2/1 | 2.86 (0.64-12.74)  1.87 (0.66-5.26)  1.53 (0.29-7.89) | 0.21 | - |  |
| Time to transplant | 0.96 (0.88-1.04) | 0.37 | - |  |
| Donor relation  (MUD/MRD) | 0.67(0.25-1.76) | 0.42 | - |  |
| HLA match vs mismatch | 0.47 (0.11-2.09) | 0.36 | - |  |
| PB vs BM graft | 0.46 (0.15-1.38) | 0.19 | - |  |
| Type of conditioning RIC vs MAC | 0.63 (0.27-1.51) | 0.29 | - |  |
| CMV match vs mismatch | 1.79 (0.69-4.65) | 0.21 | - |  |
| ABO mismatch  (match vs major mismatch | 0.89 (0.29-2.83) | 0.87 | - |  |
| HB at Day 0 | 1.00 (0.74-1.35) | 0.96 | - |  |
| WBC at Day 0 | 1.04 (0.99-1.07) | 0.06 | - |  |
| ANC at Day 0 | 1.05 (0.99-1.09) | 0.05 | - |  |
| Platelet at Day 0 | 0.99 (0.99-1.01) | 0.84 | - |  |
| Palpable splenomegaly at Day 0 | 2.06 (0.69-6.17) | 0.23 | - |  |
| Acute GVHD | 0.85(0.34-2.14) | 0.73 | - |  |
| Chronic GVHD | 0.64 (.24-1.73) | 0.39 | - |  |

*HCT CI groups: Group 1 (HCT CI = 0), Group 2 (HCT CI= 1, 2), Group 3 (HCT CI = 3 or more)

PD, progressive disease, OMR, optimal marrow response, CR, complete response

| **Table 10S: Univariate and Multivariate post-transplant LFS analysis of 46 patients who underwent allogeneic hematopoietic cell transplantation in chronic phase CMML.** | | | | |
| --- | --- | --- | --- | --- |
|  | **Univariate analysis for post-transplant LFS** | | **Multivariate analysis for post-transplant LFS** | |
| **Variables** | **HR for LFS (95% CI)** | **P value** | **HR for LFS (95% CI)** | **P value** |
| Age at Transplant | 0.99 (0.96-1.04) | 0.96 | - |  |
| Gender (M/F) | 1.19 (0.48-2.95) | 0.71 | - |  |
| PB blast %; median (range) | 1.03(0.89-1.15) | 0.61 | **-** |  |
| BM blast %; median (range) | 1.05(0.96-1.13) | 0.30 | **-** |  |
| WHO FAB subtype  (Dysplastic/Proliferative) | 1.16 (0.49-2.74) | 0.73 | - |  |
| Abnormal cytogenetics  (Abnormal/Normal) | **2.78 (1.18-6.58)** | **0.02** | **2.73 (1.04-7.17)** | **0.02** |
| Mayo French cytogenetic risk group  (Low/Intermediate)  Intermediate/High) | 0.40 (0.14-1.16)  0.82 (0.22-3.11) | 0.09 | - |  |
| Mutations:   1. *TET2* 2. *DNMT3A*   *(+/-)*   1. *IDH1* 2. *IDH2* 3. *ASXL1* 4. *EZH2* 5. *RUNX1* 6. *BCOR* 7. *SF3B1* 8. *SRSF2* 9. *U2AF1* 10. *ZRSR2* 11. *JAK2* 12. *CBL* 13. *KRAS* 14. *NRAS* 15. *CSF3R* 16. *C-KIT* 17. *FLT3* 18. *TP53* 19. *SETBP1* | 7.75 (0.81-74.5)  --  --  0.81(0.19-3.41)  --  --  --  --  0.30 (0.04-2.44)  0.69 (0.08-5.65)  --  --  --  --  4.48 (1.05-19.10)  --  --  --  4.08 (0.47-35.15)  -- | 0.09  0.14  --  0.50  0.77  0.12  0.15  -  -  0.19  0.71  0.50  0.56  0.95  0.39  0.06  -  0.30  0.39  0.27  0.83 | - |  |
| Therapy related  (Yes/No) | 0.66 (0.09- 5.04) | 0.67 | - |  |
| HMA therapy before transplant(Yes/No) | 0.82 (0.32-2.07) | 0.47 | - |  |
| AML induction like chemotherapy before transplant(Yes/No) | 1.75 (0.59-5.22) | 0.34 | - |  |
| Disease status at transplant  PD/OMR  OMR/CR | 2.24 (0.70-7.15)  1.28 (0.25-6.63) | 0.36 | - |  |
| HCT CI groups*  3/1  3/2  2/1 | 2.67 (0.59-11.88)  1.83 (0.65-5.15)  1.46 (0.28-7.54) | 0.25 | - |  |
| Time to transplant | 0.96 (0.87-1.04) | 0.34 | - |  |
| Donor relation  (MUD/MRD) | 0.65 (0.24-1.71) | 0.38 | - |  |
| HLA match vs mismatch | 0.53 (0.12-2.31) | 0.43 | - |  |
| PB vs BM graft | 0.45 (0.15-1.38) | 0.19 | - |  |
| Type of conditioning RIC vs MAC | 0.62 (0.26-1.47) | 0.27 | - |  |
| CMV match vs mismatch | 1.86 (0.71-4.80) | 0.19 | - |  |
| ABO (match vs major mismatch | 0.84 (0.27-2.63) | 0.86 | - |  |
| HB at Day 0 | 1.00 (0.74-1.34) | 0.99 | - |  |
| WBC at Day 0 | 1.06 (1.01-1.11) | **0.01** | 0.94 (0.61-1.43) | 0.69 |
| ANC at Day 0 | 1.08 (1.02-1.13) | **0.01** | 1.16 (0.71-1.87) | 0.47 |
| Platelet at Day 0 | 0.99 (0.99-1.01) | 0.82 | - |  |
| Palpable splenomegaly at Day 0 (present/absent) | 2.02 (0.67-6.07) | 0.24 | - |  |
| Acute GVHD (Y/N) | 0.90 (0.36- 2.27) | 0.82 | - |  |
| Chronic GVHD (Y/N) | 0.67 (0.25-1.79) | 0.43 | - |  |

*HCT CI groups: Group 1 (HCT CI = 0), Group 2 (HCT CI= 1, 2), Group 3 (HCT CI = 3 or more)

PD, progressive disease, OMR, optimal marrow response, CR, complete response.

**Supplementary Methods:**

Neutrophil engraftment was defined as the first of 3 consecutive days of ANC ≥0.5 x 109/ L and platelet engraftment was defined as the first of 7 days of a sustained platelet count of ≥ 20x109/L without requiring transfusions. AML like induction chemotherapy constituted the standard 7+3 regimen with either daunorubicin or idarubicin, and cytarabine. Two patients in the chronic phase received cladribine and 1 patient received imatinib. The 3 patients enrolled in clinical trials received either tipifarnib and tagraxofusp , or lenzilumab or MEK 1 and 2 inhibitor mirdametinib.

Myeloablative conditioning regimens were either Busulfan (12.8 mg/kg, IV) /Cyclophosphamide (120mg/kg, IV) or TBI-based (1200 cGy)-based regimens, whereas Fludarabine (125 mg/m2) /Melphalan (140 mg/m2 IVx1) and Fludarabine (150 mg/m2) /Busulfan (6.4 mg/kg, IV) were the preferred reduced intensity conditioning regimens. Immunosuppressive regimens for GVHD prophylaxis were combinations of methotrexate at 5 and 10 mg/m2 and calcineurin inhibitors cyclosporine or tacrolimus.

Kaplan-Meier estimates of overall Survival (OS) and OS post-transplant were calculated from the date of diagnosis and transplant date until the date of death respectively or censored at last documented follow-up. In the calculating LFS, transformation to AML replaced death as the uncensored event. Statistical comparisons were performed using the log-rank test. Univariate and multivariate survival analyses were performed using Cox logistic regression analysis. Patients with more than 20% blasts in the bone marrow were considered to be in AML or blast transformation (BT).

Acute and chronic graft versus host disease (GVHD) was defined as per the Glucksberg grading and NIH consensus criteria 2014 respectively. The composite end point of GVHD-free/relapse-free survival (GRFS) was calculated which incorporates grade 3-4 acute GVHD, chronic GVHD requiring systemic treatment, relapse or death in the first year following HCT.
